# Single‐Cell Sequencing Data Revealed Mechanisms of Interactions Between Tumor Cells and Cancer‐Associated Fibroblasts in Metastatic Colorectal Cancer

**DOI:** 10.1155/bmri/8830690

**Published:** 2026-03-13

**Authors:** Yiqing Tan, Yiping Yang, Fuhua Tian, Ni Li, Lei Hu, Mingyou Deng, Yingying Wang, Zuwei Xia, Ran Sun

**Affiliations:** ^1^ Department of Breast Surgery, Sichuan Academy of Medical Sciences & Sichuan Provincial People’s Hospital, School of Medicine, University of Electronic Science & Technology of China, Chengdu, China, samsph.com; ^2^ Key Laboratory of Molecular Oncology and Epigenetics, The First Affiliated Hospital of Chongqing Medical University, Chongqing, China, cqmu.edu.cn; ^3^ Department of Oncology, Jiulongpo People’s Hospital, Chongqing, China

**Keywords:** cancer-associated fibroblasts, metastatic colorectal cancer, prognosis, single-cell sequencing, tumor cell

## Abstract

Colorectal cancer (CRC) is highly metastatic, yet the interaction between tumor cells and microenvironment components remains unclear, as does its impact on patient outcomes. Single‐cell data (GSE225857 and GSE166555) were collected for transformed and primary CRC to identify differing cell types and their subpopulations. Prognostic signature genes for CRC were identified through Cox analysis of tumor cell subpopulations and cancer‐associated fibroblasts (CAFs) interaction genes, leading to the development of a prognostic model. In the single‐cell dataset of metastatic CRC patients in GSE225857 and nonmetastatic CRC patients in GSE166555, eight tumor cell subpopulations were identified, in which T0 was significantly enriched in metabolism‐associated pathways. The CAFs subpopulation CAF0 and T0 had extensive cell communication and were approachable in three‐dimensional space. A prognostic signature predicting CRC patient survival was developed and validated based on these signature genes of CAF0 cells. This prognostic signature serves as an independent and effective factor for prognosis. In this study, we identified characteristic cell subpopulations in metastatic and primary CRC. Based on the reciprocal genes between them, we constructed a prognostic model for CRC. Our findings provide a scientific basis for understanding the metastatic mechanisms of CRC.

## 1. Introduction

Colorectal cancer (CRC) is one of the common gastrointestinal malignant tumors. According to the global cancer statistics report, CRC ranked third in the number of new cases and deaths worldwide in 2025 [1]. Cancer statistics in the United States for the last 3 years have reported a surge in the projected number of new cases of CRC, with 25% of patients found to have developed liver metastases [2–4]. There are numerous risk factors for CRC, which include family history, gender, smoking, alcohol abuse, physical inactivity, obesity, and red and processed meat consumption [5]. Accompanied by improvements in early screening and diagnosis and various therapeutic approaches, the survival time of CRC has been extended compared to the previous period, but the overall survival (OS) rate in the high‐risk stage has not improved significantly [6]. At present, the 5‐year OS rate of CRC patients in China is 50%–60%, and the treatment effect for CRC patients still needs to be improved [7]. Therefore, it is necessary to further explore the pathogenesis of CRC and search for new potential biomarkers and therapeutic targets for the treatment of CRC.

CRC is highly heterogeneous, and traditional research methods are only able to analyze the whole tumor tissue and are unable to compare intratumor differences at the single‐cell level [8]. Single‐cell sequencing technology (scRNA‐seq) can provide a more in‐depth study of cell heterogeneity by targeting individual cells. scRNA‐seq combined with high‐throughput sequencing technology makes it possible to resolve the heterogeneous molecular features of tumor tissues, which can be applied to basic research, clinical diagnosis, and drug development [9]. In addition, it can be used to discover abnormally proliferating cell types to search for new pathogenesis of CRC [10]. scRNA‐seq can also reveal CRC liver metastasis features [11].

Cancer‐associated fibroblasts (CAFs) are the major stromal cell component in most solid tumors, including breast cancer, prostate cancer, and pancreatic cancer [12, 13]. CAFs also coexist as a heterogeneous population. Several CAF subtypes with distinct molecular characteristics have been identified in various cancers [14–19], but the identification of all potential subtypes and their distinct functional roles remain incomplete. scRNA‐seq enables the analysis of gene expression profiles of individual cells in tissues with complex structures and provides us with a high‐resolution window to gain deeper insights into transcriptional differences. In turn, these molecular differences may help us better understand the functions of each specific cell type [20]. Moreover, scRNA‐seq enables the identification of rare cell populations that may remain undetected using traditional methodologies [21]. Several studies have utilized scRNA‐seq to explore the heterogeneity of CAFs in solid tumors, thereby enhancing our understanding of the heterogeneity of CAFs [12, 18, 19]. However, the subpopulations of CAFs and their interactions with tumor cells remain unclear at present.

In this study, by analyzing primary CRC tumor samples and metastatic CRC samples in a dual dataset, we carved out the cell types in metastatic CRC samples that are different from those in primary CRC samples. A prognostic model for CRC was then developed based on the prognostic signature genes associated with the interactions of these cell types. Our study revealed important cell types and potential mechanisms in CRC metastasis.

## 2. Materials and Methods

### 2.1. Dataset Information

FPKM expression profiles (log transformed), survival information (OS), and clinical information for TCGA‐COAD and TCGA + READ were downloaded from the University of Cingifornia Sisha Cruz Xena (UCSC Xena) website (https://xenabrowser.net/), and both expression and survival information were retained for 606 tumor samples for signature construction. Among all the available information, 606 tumor samples with both expression and survival information were retained for signature construction. Two sets of bulk expression profiles, GSE17534 and GSE39582, and corresponding clinical information were downloaded from the Gene Expression Omnibus (GEO) database (https://www.ncbi.nlm.nih.gov/geo/) and used for signature validation. Data processing standard: According to the probe correspondence of each platform, the probes were converted into symbols; if a probe corresponded to multiple genes, the probe was removed; if multiple probes corresponded to the same symbol, the median value was taken. The expression profiles (UMI‐count) of single‐cell datasets with registration numbers GSE225857 and GSE166555 were downloaded from the GEO database, and 6 CRC patients with metastasis in GSE225857 and 12 CRC patients without metastasis in GSE166555 were selected for the analysis of this project. Clinical data and transcriptomic data from the cancer patient cohorts treated with PD‐1/PD‐L1 blockers in GSE78220 and GSE42664 were downloaded from the GEO database and used to assess the predictive efficacy of the signature in immunotherapy cohorts.

### 2.2. Single‐Cell Transcriptome Data Quality Control (QC)

A total of 18 samples from the GSE225857 and GSE166555 datasets were QC using the R package Seurat (v4.3.1) [22], and in order to exclude low‐quality cells and lowly expressed genes, we applied the following thresholds: (1) each gene is expressed in no fewer than three cells; (2) the number of features per cell exceeds 200, and the number of counts per cell exceeds 200; and (3) the percentage of mitochondrial genes as well as the percentage of erythroid genes in each cell is less than 40%. Then, the NormalizeData function was used for normalization, and the vst function in FindVariableFeatures was used to identify highly variable genes. Batch correction between samples was performed by the R package harmony [23] to avoid batch effects interfering with swim analysis. We then scale‐transformed the data, performed dimensionality reduction using principal component analysis, and selected the Top 20 principal components for downstream analysis. Cell annotation information (major cell types as well as fibroblast subpopulations) was performed for this analysis using the dataset‐supplied as well as classical markers.

### 2.3. Tumor Cell Subpopulation Identification

Based on the meta information of the original article (epithelial cells from which tumor samples originate are malignant tumor cells), cells annotated as malignant cells (epithelial cells from which tumors originate) were extracted from 18 tumor samples, which were normalized, identified high variant genes, removed from batch effect, and PCA. We selected the first 20 principal components and set resolution = 0.1 and again clustered and binned to identify 8 subpopulations of tumor cells. The characteristic genes of each subpopulation of cells were identified by FindAllMarkers (avg_log2fc > 1, *p*_val_adj < 0.05).

### 2.4. Cell Spatial Organization and Analysis of Cell Communication

The spatial organization of cells is closely associated with a range of cellular functions and behaviors, particularly cell–cell interactions. However, this spatial information is typically lost in scRNA‐seq data due to the requirement for cell dissociation prior to sequencing. To address this limitation, we employed the CSOmap algorithm [24] to reconstruct spatial gene expression patterns solely from scRNA‐seq data. CSOmap enables the prediction of cell–cell interactions, the inference of cellular spatial organization, the construction of spatial expression profiles of ligands and receptors, and the identification of intercellular communication networks. Furthermore, it facilitates the elucidation of signaling mechanisms underlying key biological processes such as tumor development, progression, and cellular differentiation. In this study, the CSOmap algorithm was applied to infer three‐dimensional spatial accessibility and communication signals between tumor cell subpopulations and fibroblasts. To further investigate potential interaction dynamics between tumor cells and fibroblasts in CRC, we conducted a comprehensive cell–cell communication analysis between tumor cell subpopulations and fibroblast subpopulations using the R package CellChat [25].

### 2.5. Construction and Validation of Prognostic Markers Based on Cell Interaction Mechanisms

Based on the characteristic genes of tumor cell subpopulations, the mean expression levels of these genes were calculated for each subpopulation in TCGA‐COAD and READ samples to define subpopulation‐specific signatures. The prognostic value of each subpopulation signature was subsequently evaluated using univariate Cox proportional hazards regression analysis to determine the associated hazard ratios (HRs) and their statistical significance.

Fibroblast subpopulations (CAF0) with significant interactions with prognostically relevant tumor cell subpopulations (T0, *p* < 0.05) and spatially accessible subpopulations were selected by combining cell communication and spatial communication information. The prediction of CAF0 subpopulation (vs. other fibroblast subpopulations) by CAF0 subpopulation signature genes was evaluated by receiver operating characteristic (ROC), and area under the curve (AUC) was visualized using the R package pROC [26]. Genes with AUC > 0.7 were screened, and the significance score of different CAF subgroups for each sample was calculated using the mean value algorithm. Samples were divided into two groups of high and low according to the score, and survival curves for prognostic analyses were generated by the Kaplan–Meier, and the significance of the differences was determined using the log‐rank test to further parse out the correlation of these two types of samples with the OS correlation. In addition, univariate and multivariate Cox analyses were conducted to evaluate the independent prognostic value of score.

### 2.6. Gene Set Variation Analysis (GSVA)

GSVA is a nonparametric, computational method widely used to estimate pathway and biological process activity across samples. Gene sets from three sublibraries, HALLMARK, KEGG, and GO biological process (GOBP), were obtained from the Molecular Signatures Database (MSigDB, https://www.gsea-msigdb.org/gsea/msigdb) and applied in GSVA to assess functional enrichment patterns. Differences in the function of different tumor cell subtypes were compared using the R package GSVA [27] and GSEABase.

### 2.7. Statistical Analysis

All analyses were conducted using R software Version 4.1.2. The Wilcoxon rank‐sum test was employed to assess differences between two groups, while the Kruskal–Wallis test was used for comparisons among multiple groups. These analyses were applied to various variables, including gene expression levels, infiltration percentages, and other eigenvalues. Statistical significance is denoted as follows: ns *p* > 0.05;  ^∗^ *p* < 0.05;  ^∗∗^ *p* < 0.01;  ^∗∗∗^ *p* < 0.001; and  ^∗∗∗∗^ *p* < 0.0001. Survival analysis was generated using the Kaplan–Meier and log‐rank test.

## 3. Results

### 3.1. Cell Mapping in COAD Tumor Tissue

The cell data in GSE225857 and GSE166555 were processed by the Seurat code package and retained for 94,499 cells. These cells were analyzed by clustering and annotated with marker genes and could be identified into eight cell types: plasma, B cells, endothelial cells, tumor cells, fibroblasts, MAST cells, myeloid cells, and T/NK cells (Figures 1a, 1b, 1c, and 1d). We counted the number and proportion of various cell types in metastatic and primary tumor samples. Compared to primary tumor samples, the proportion of fibroblasts was increased, and the proportion of tumor cells was decreased in metastatic samples (Figure 1e). These results suggest that an increased proportion of fibroblasts may promote the occurrence of distal metastasis of tumor cells.

Figure 1Cell mapping in COAD tumor tissue. (a) Distribution status of cells in samples in GSE225857 and GSE166555 before removal of batch effects. (b) Distribution status of cells in samples from GSE225857 and GSE166555 after removal of batch effects. (c) Clustering plot of the eight cell types after marker genes annotation. (d) Expression bubble diagram of marker genes in eight cell types. (e) Proportion of eight cell types in primary and metastatic tumors.

**Figure:**
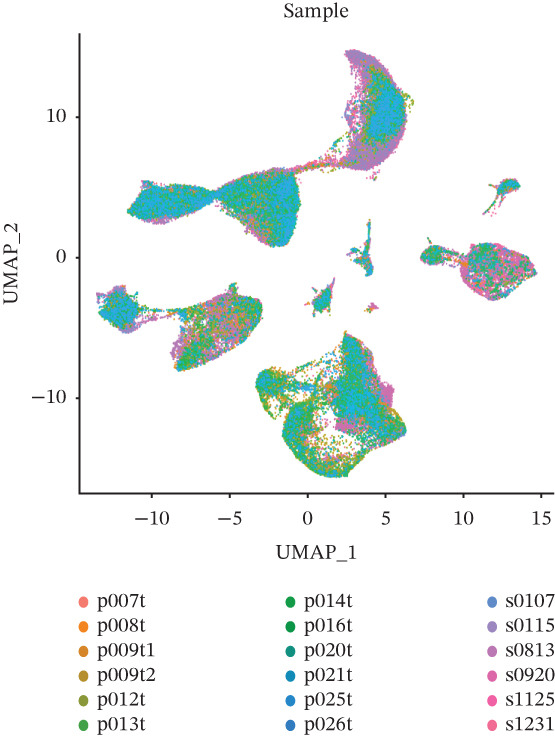
(a)

**Figure:**
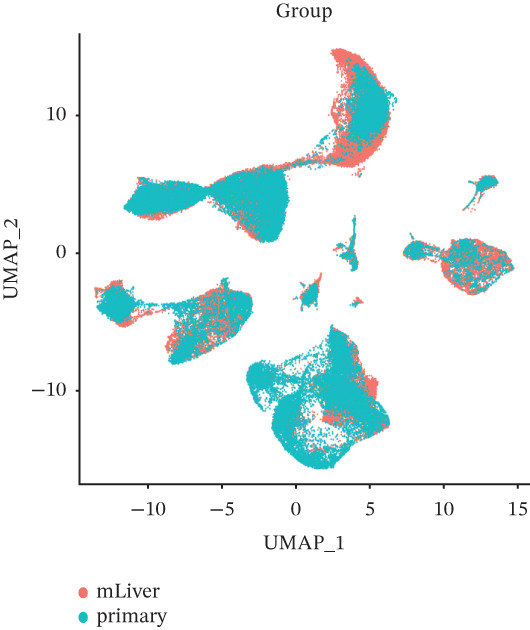
(b)

**Figure:**
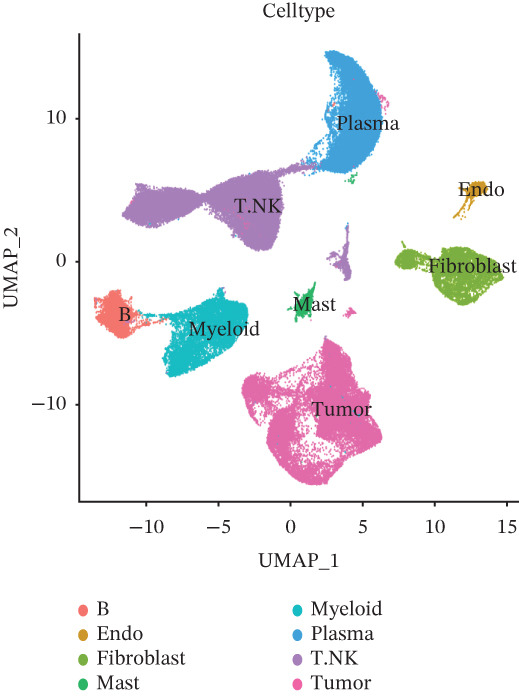
(c)

**Figure:**
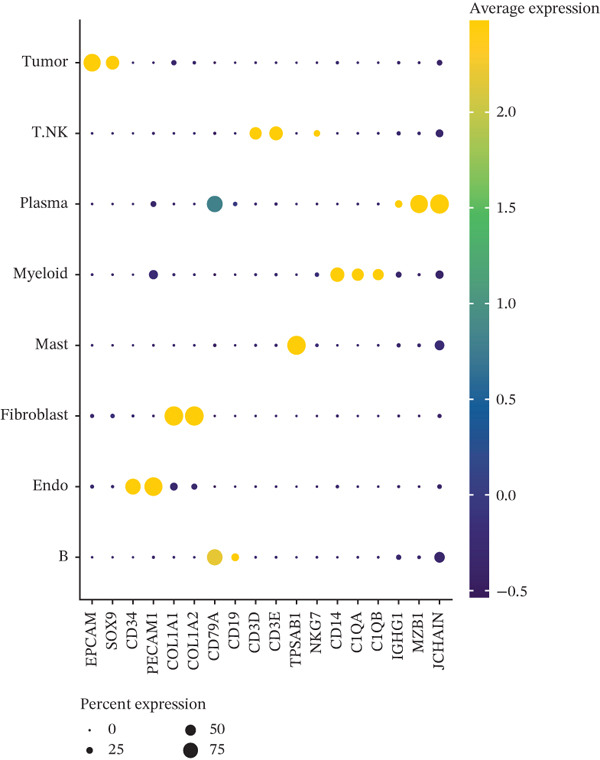
(d)

**Figure:**
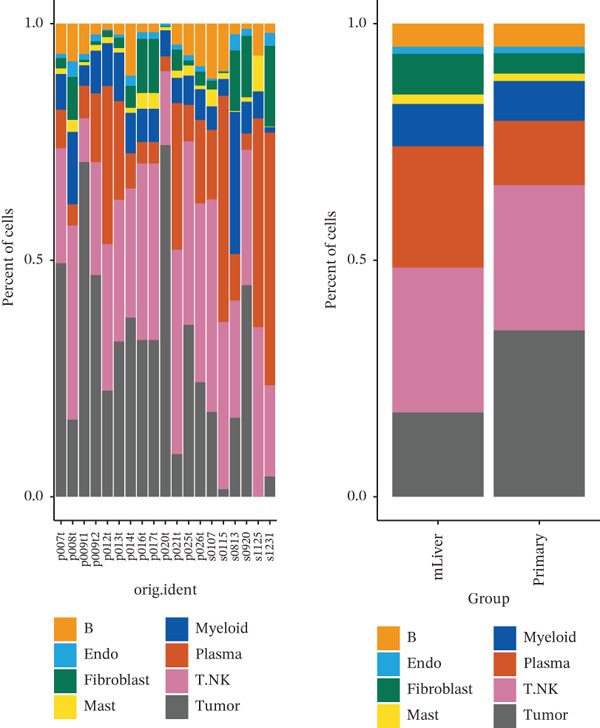
(e)

### 3.2. Tumor Cell Subpopulation Landscape

Tumor cells in the extracted double dataset were further removed from which batch effects were present, and their cell data were subsequently normalized and clustered into subpopulations (Figure 2a). We found that there were eight cell subpopulations in tumor cells: Cycling_T2, T0, T1, T3, T4, T5, T6, and T7 (Figure 2b). The eight cell subpopulations were analyzed differentially to identify the differentially expressed genes (DEGs) among them. We showed the Top 10 DEGs with the highest expression among the 8 cell subpopulations (Figure 2c). Interestingly, cell cycle pathway‐related genes were significantly highly expressed in Cycling_T2, including TOP2A, MKI67, CENPF, HMGB2, STMN1, TUBA1B, HMGN2, CCNB1, and PTTG1 (Figure 2d). We analyzed the biological pathways related to different cell subpopulations. Cycling_T2 was mainly involved in DNA replication, nucleocytoplasmic transport, spliceosome, and cell cycle pathways (Figure 2e). We selected the Top 3 most prominent pathways for visualization, and cell cycle, motor proteins, and spliceosome were the pathways that were significantly activated in the Cycling_T2 subgroup (Figure 2f). T0 is mainly involved in Parkinson’s disease, PI3K‐Akt signaling pathway, and pathways in cancer pathway (Figure 2g). We also selected the Top 3 most prominent pathways for visualization, and amoebiasis, coronavirus disease‐COVID‐19, and ribosome were the pathways that were significantly activated in the T0 subgroup (Figure 2h).

Figure 2Tumor cell subpopulation landscape and biological pathways involved in Cycling_T2 and T0. (a) Distribution status of tumor cells in samples from GSE225857 and GSE166555 before removal of batch effects. (b) Distribution of eight cell subpopulations in tumor cells: Cycling_T2, T0, T1, T3, T4, T5, T6, and T7. (c) Heat map of the expression of the Top 10 DEGs in eight cell subpopulations. (d) Expression bubble diagrams of cell cycle pathway‐related genes in eight cell subpopulations. (e) Bubble diagram of biological pathways enriched in the Cycling_T2 subpopulation. (f) Map of the Top 3 biological pathways enriched in the Cycling_T2 subpopulation. (g) Bubble diagram of biological pathways enriched in the T0 subpopulation. (h) Map of the first three biological pathways enriched in the T0 subpopulation.

**Figure:**
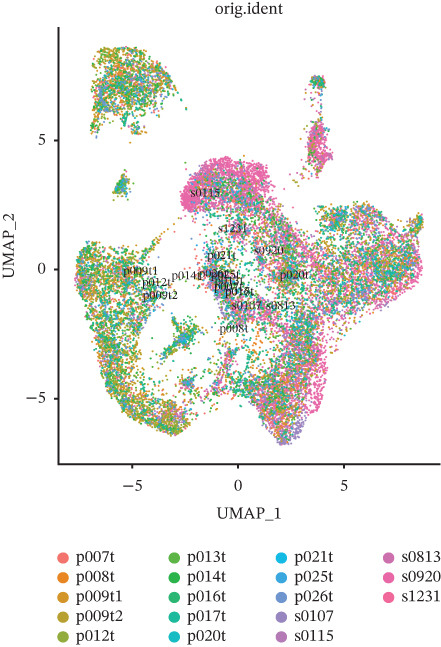
(a)

**Figure:**
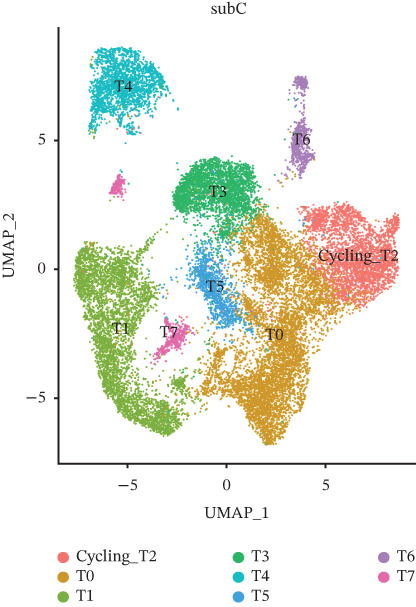
(b)

**Figure:**
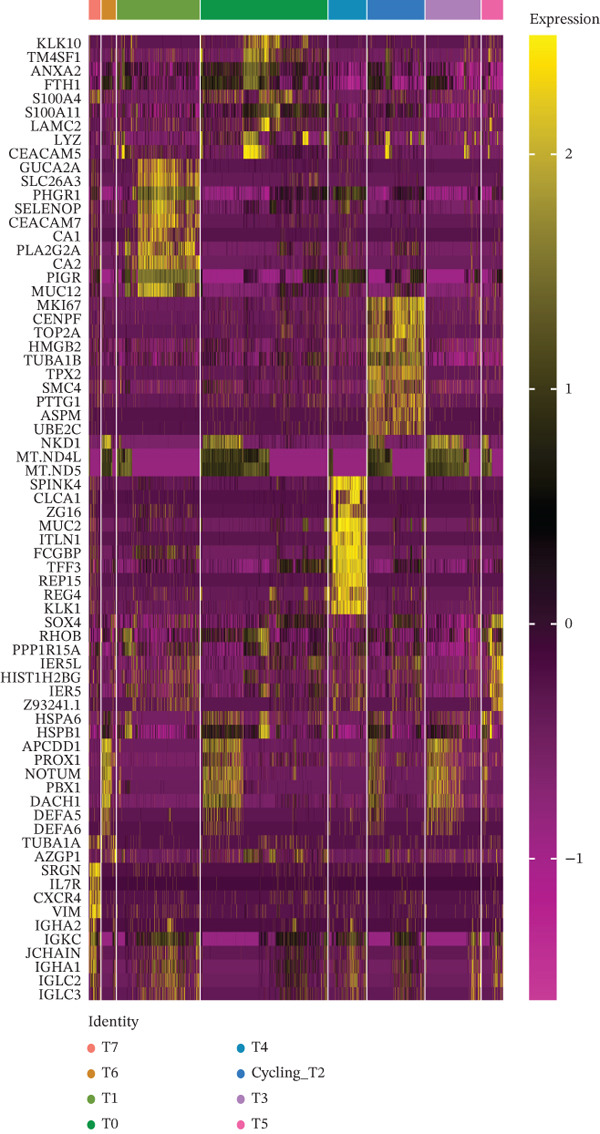
(c)

**Figure:**
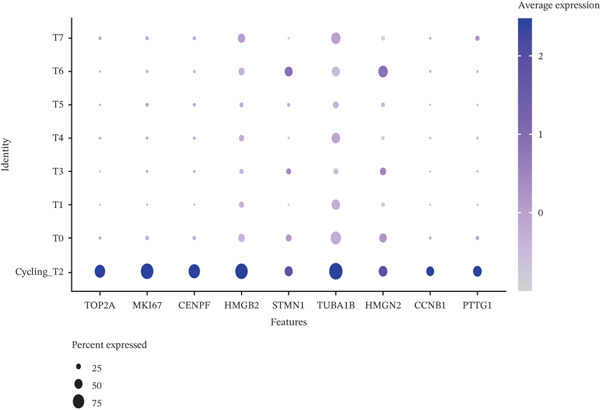
(d)

**Figure:**
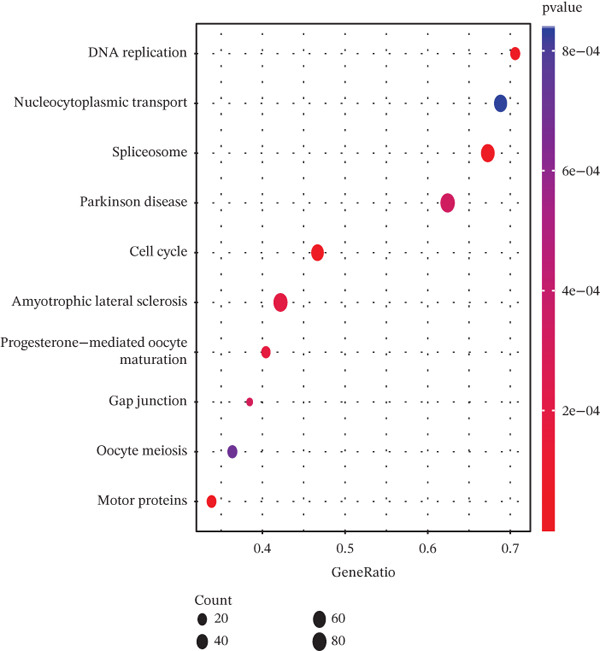
(e)

**Figure:**
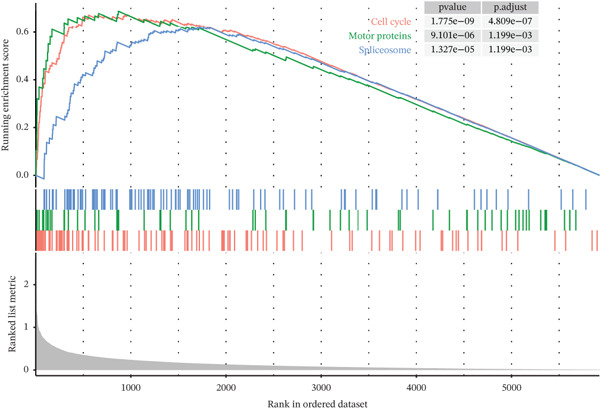
(f)

**Figure:**
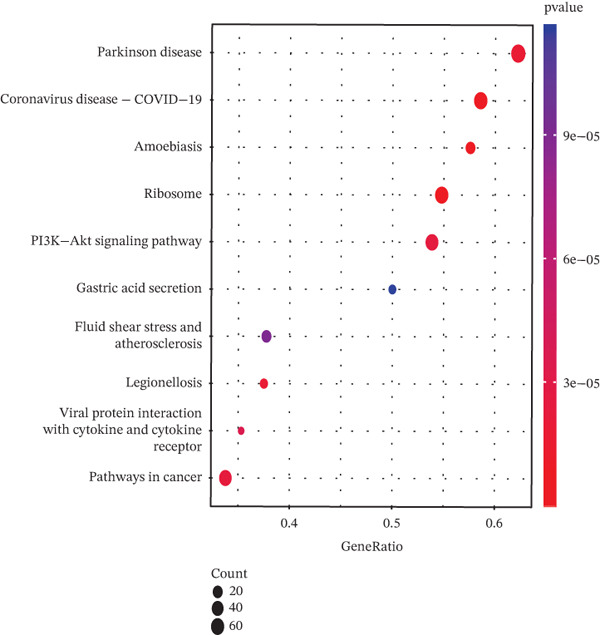
(g)

**Figure:**
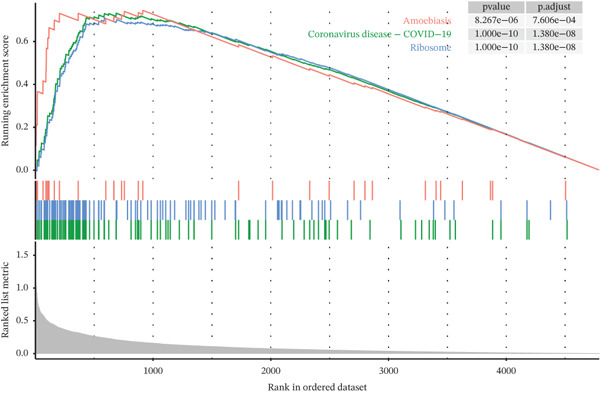
(h)

### 3.3. Fibroblast Subgroup Landscape

As in the previous results, we found that an increased proportion of fibroblasts may promote tumor cells to undergo distal metastasis. We extracted all the fibroblasts data in the double dataset and firstly removed their batch effect (Figure 3a). Then, the fibroblast data were normalized and analyzed by cell clustering. Based on the CAF‐related genes annotating these cell subclusters, nine CAF subclusters, CAF0, CAF1, CAF2, CAF3, CAF4, CAF5, CAF6, CAF7, and CAF8, existed in fibroblasts (Figure 3b). Based on the differential analysis, we found the Top 10 expressed DEGs in the 9 subpopulations (Figure 3c). CAF1 was the high percentage cell subpopulation in metastatic COAD, and CAF0 and CAF2 were the high percentage cell subpopulations in primary COAD (Figure 3d). In addition, sets of genes in the HALLMARK_ANGIOGENESIS, NEGATIVE_REGULATION_OF_ANGIOGENESIS, REGULATION_OF_ANGIOGENESIS, and WP_ANGIOGENESIS pathways were downloaded, and we assessed their nine subpopulations in terms of their expression levels (Figure 3e). COL1A1 and COL1A2 were highly expressed in CAF1, and THY1 was highly expressed in CAF0 (Figure 3e). Based on the differential expression results, the CAF0 subpopulation mainly expressed SFRP2 and SFRP4 (Table 1). The expression levels of the remaining genes in the four pathways across the nine subpopulations are presented as heat maps (Figure S1).

Figure 3Fibroblast subpopulation landscape and signature genes in the angiogenesis factor‐related pathway in nine CAF subpopulations. (a) Landscape of CAF subpopulations. (b) The nine CAF subgroups in the sample. (c) Heat map of DEG expression in nine CAF subpopulations. (d) Proportion of nine CAF subgroups in metastatic COAD and primary COAD. (e) Expression levels of CAF signature genes in nine CAF subpopulations.

**Figure:**
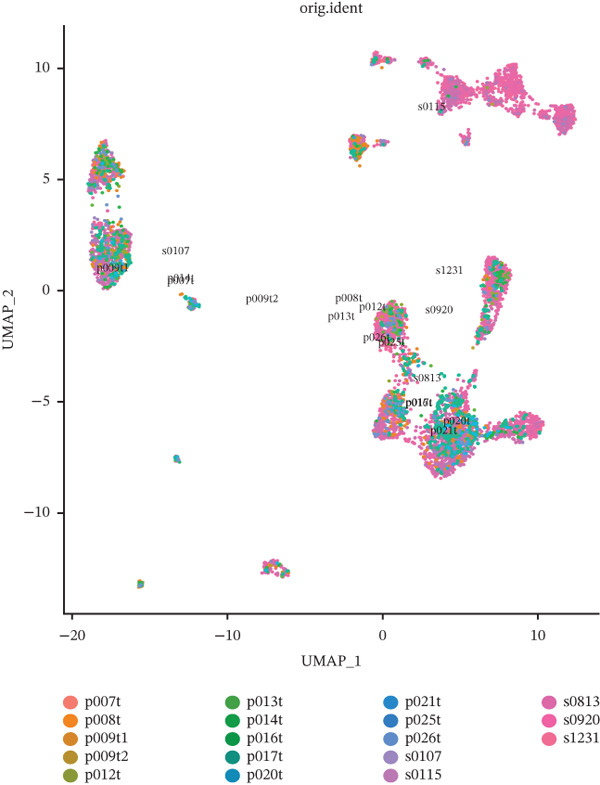
(a)

**Figure:**
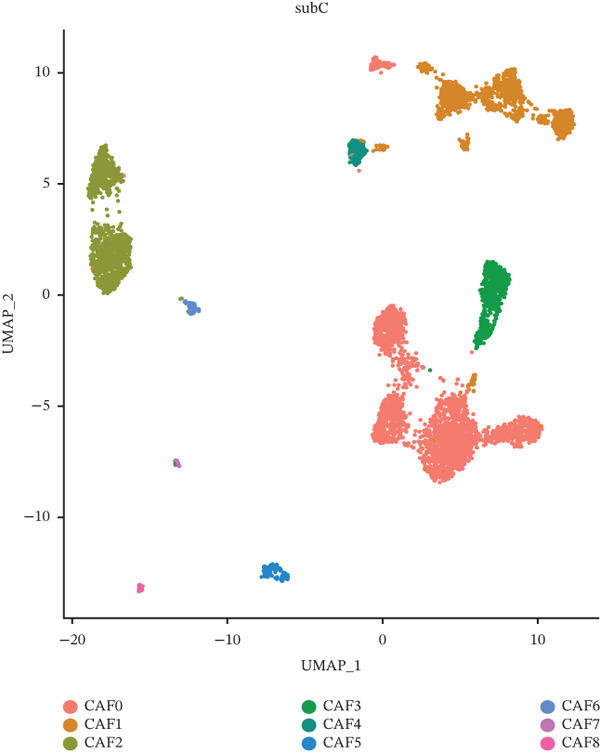
(b)

**Figure:**
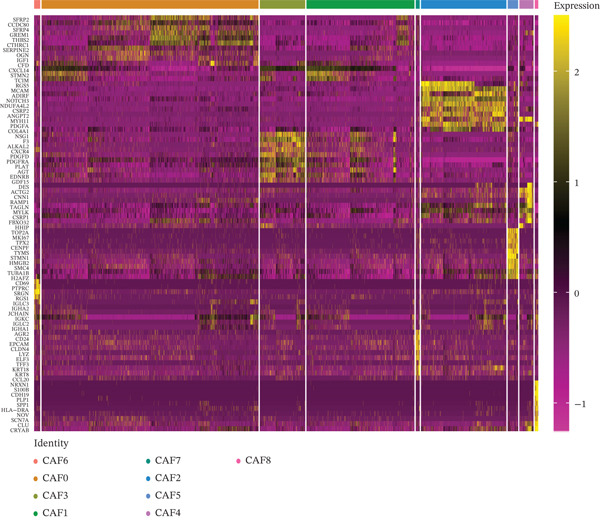
(c)

**Figure:**
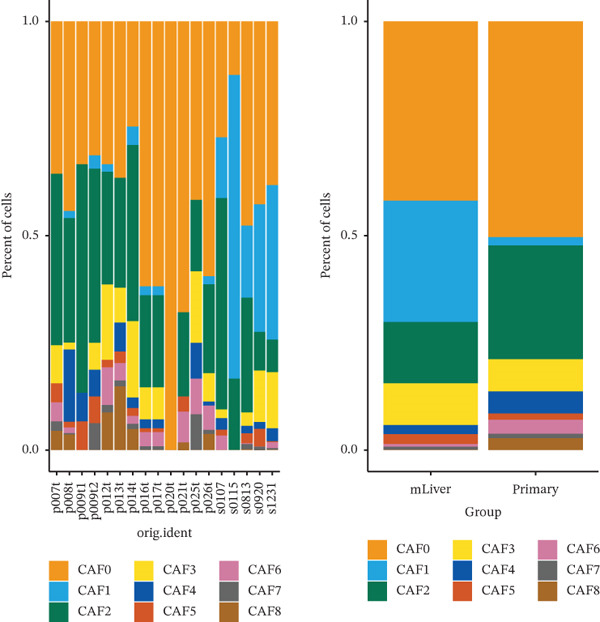
(d)

**Figure:**
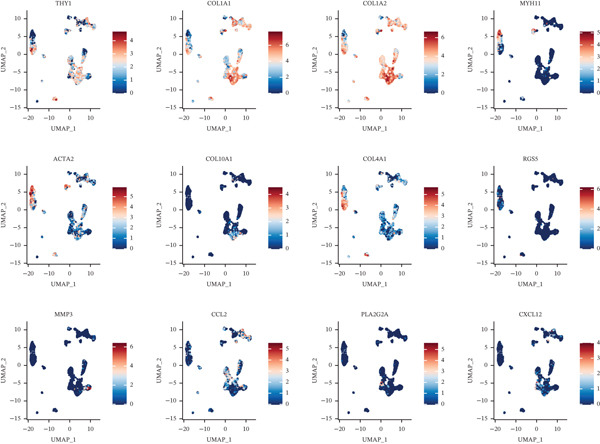
(e)

**Table 1:** Six signature genes in CRC prognostic models.

**Gene**	**p** **_value**	**Avg_log2FC**	**p** **_val_adj**	**Cluster**
LUM	0	1.297445176	0	CAF0
DCN	2.38e−287	1.084834897	5.46e−283	CAF0
SFRP2	3.89e−261	2.572351318	8.93e−257	CAF0
CCDC80	2.96e−251	1.564961552	6.78e−247	CAF0
SFRP4	4.45e−239	2.157019344	1.02e−234	CAF0
GREM1	3.26e−228	1.690360442	7.47e−224	CAF0

### 3.4. Analysis of Cell Communication Between Tumor Cells and CAFs

CAFs can secrete a variety of chemokines and growth factors that play critical roles in the tumor microenvironment and regulate tumor progression. Based on our identified tumor cells and CAFs, we performed cell communication analysis. We found a strong density and intensity of cell communication between the CAF0 subpopulation and T0, Cycling_T2 (Figure 4a), strong binding between ligands and receptors in the WNT signaling pathway between CAF0, CAF1, T0, and Cycling_T2 (Figure 4b), and strong binding between CAF0‐T1 cell‐to‐cell interactions were carried out through COL1A1‐SDC4, COL1A2‐SDC4, COL6A1‐SDC4, and FN1‐SDC4. The cell‐to‐cell interactions between CAF1‐T1 were carried out through COL1A1‐SDC4 and COL1A2‐SDC4 for cell‐to‐cell interactions (Figure 4c). In addition, we assessed the approachability of tumor cells and CAFs in terms of distance in three‐dimensional space. We found extensive interactions between subpopulations in both cell types (Figure 4d). Specifically, CAF0 cells had three‐dimensional spatial proximity to T0 and T7 subpopulations (Figure 4e).

Figure 4Analysis of cell communication between tumor cells and CAFs. (a) Strength of ligand–receptor binding in the analysis of cell communication between subpopulations of tumor cells and subpopulations of CAFs. (b) Ligand–receptor binding strength in the WNT signaling pathway between subpopulations of tumor cells and subpopulations of CAFs. (c) Strength of ligand and receptor interactions between subpopulations of tumor cells and subpopulations of CAFs. (d) Demonstration of cell distribution in 3D space. (e) Cell subpopulation accessibility results.

**Figure:**
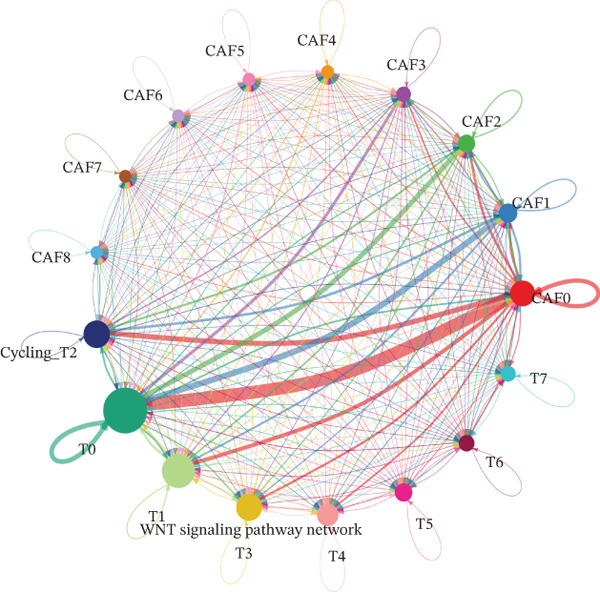
(a)

**Figure:**
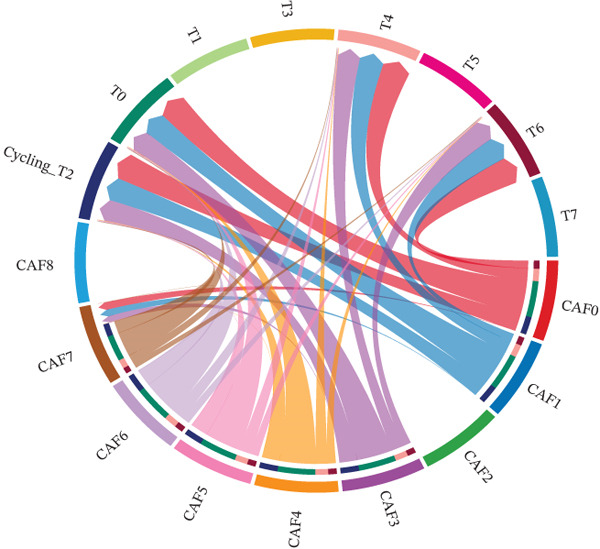
(b)

**Figure:**
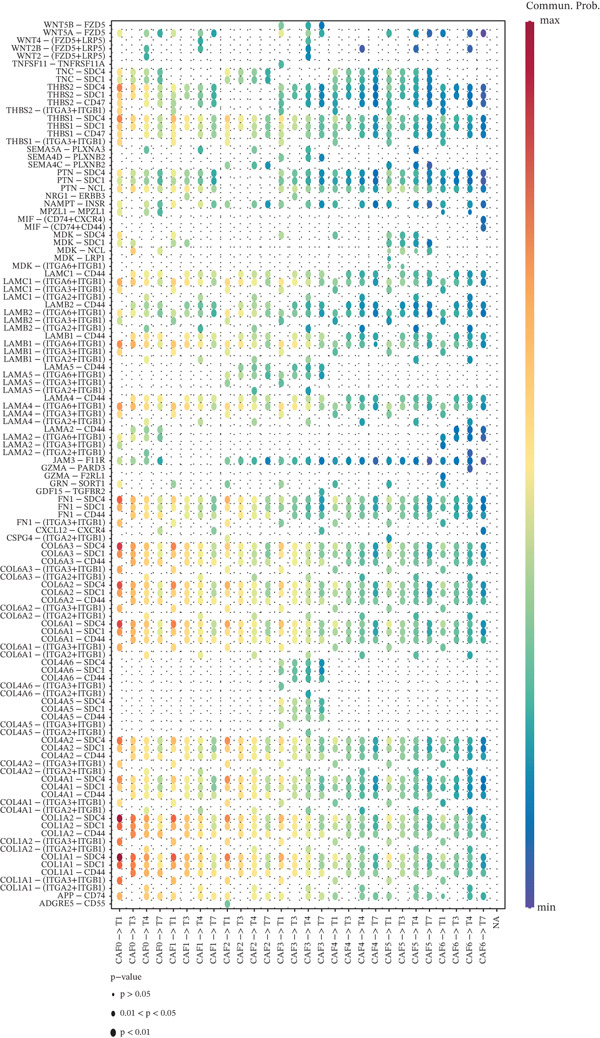
(c)

**Figure:**
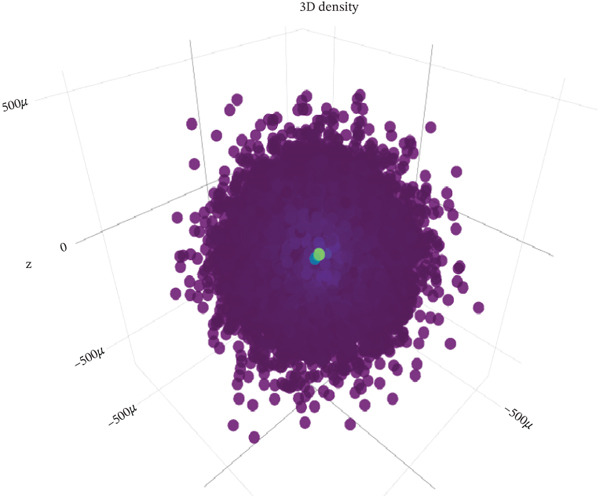
(d)

**Figure:**
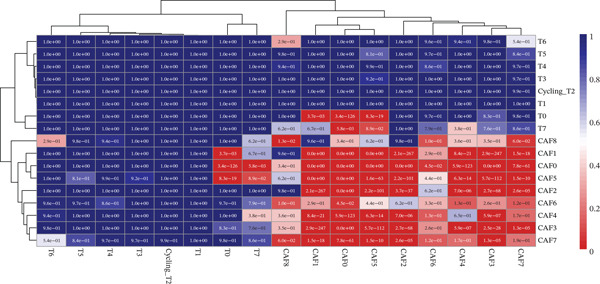
(e)

### 3.5. COAD Prognostic Modeling Based on Interaction Mechanisms Between Subpopulations of Tumor Cells and CAFs

Based on the characteristic gene expression of the tumor cell subpopulation and the CAF subpopulation, the expression mean was used to calculate the characteristic value of each sample in TCGA − READ + COAD, and the samples were divided into high and low characteristic groups according to the median, and then, the association with OS was analyzed using one‐way Cox analysis. The results showed a significant survival advantage for patients in the high median group in the T0 subgroup (Figure 5a). Next, we explored the clinical independence of the prognostic characteristics of the T0 subgroup. Multivariate Cox analysis was performed, and the results showed that the T0 subgroup prognostic trait was an independent prognostic factor for CRC (Figure 5b). By integrating the results of cell communication and three‐dimensional spatial accessibility analysis, we identified genes in the CAF0 subpopulation that exhibited significant interactions with the tumor T0 subpopulation and were spatially accessible for feature screening (*p*_val_adj < 0.05), (∣log 2FC | >1). Twenty‐nine genes were of prognostic value, among which six featured genes had predictive efficacies of the CAF0 subpopulation AUC > 0.7, and the LUM gene had the highest AUC of 0.79 (Figure 5c). We used these six signature genes as signatures of the interaction mechanism between the subpopulation of tumor cells and the subpopulation of CAFs (Exhibit 1) and calculated TCGA‐COAD + READ sample signature scores using a mean value algorithm. We found that patients with low signature scores had a significant survival advantage (Figure 5d). Similarly, a multivariate Cox analysis combining gender, stage, and age was performed, and we found that the signature score was an independent prognostic factor for CRC (Figure 5e).

Figure 5Construction of a COAD prognostic model based on the mechanism of interactions between subpopulations of tumor cells and subpopulations of CAFs. (a) K‐M survival curves based on eigenvalue scores in the T0 subgroup. (b) Forest plot of multivariate Cox analysis integrating eigenvalue scores in gender, stage, age, and T0 subgroups. (c) ROC curve for LUM. (d) K‐M curves based on K‐M curves of a signature score consisting of six characterized genes for the mechanism of interactions between subpopulations of tumor cells and subpopulations of CAFs. (e) Multivariate Cox forest plot integrating interactions between gender, stage, age, and tumor cell subgroup and CAF subgroup signature score.

**Figure:**
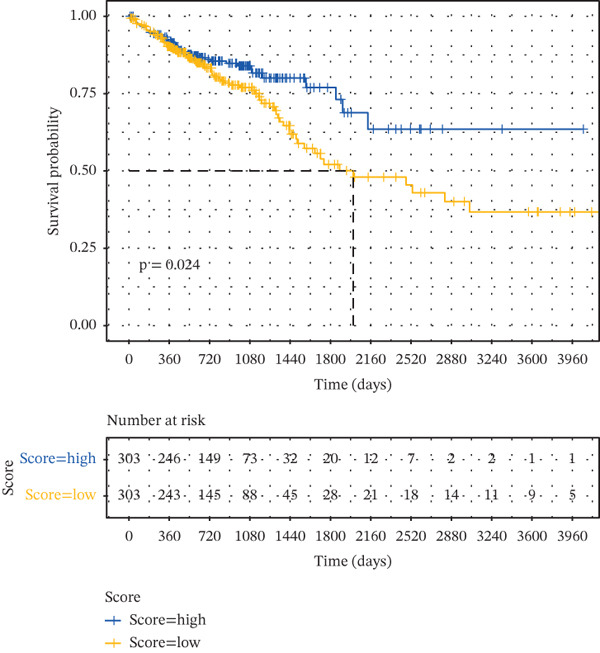
(a)

**Figure:**
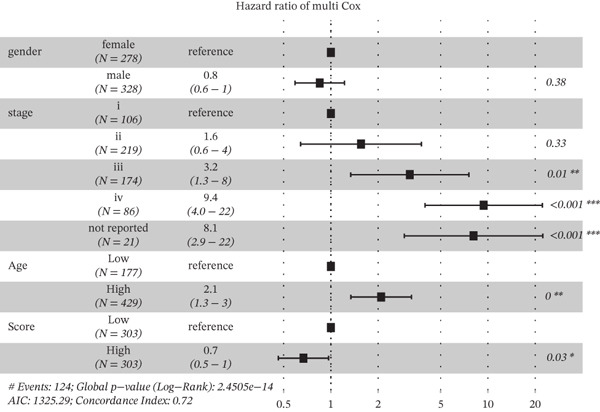
(b)

**Figure:**
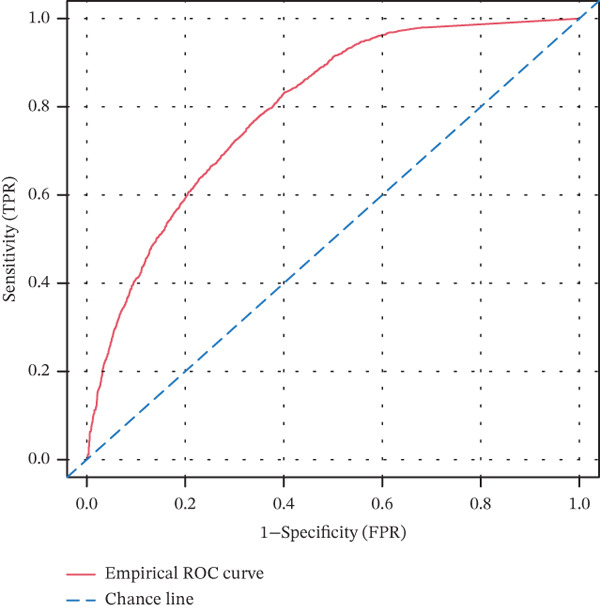
(c)

**Figure:**
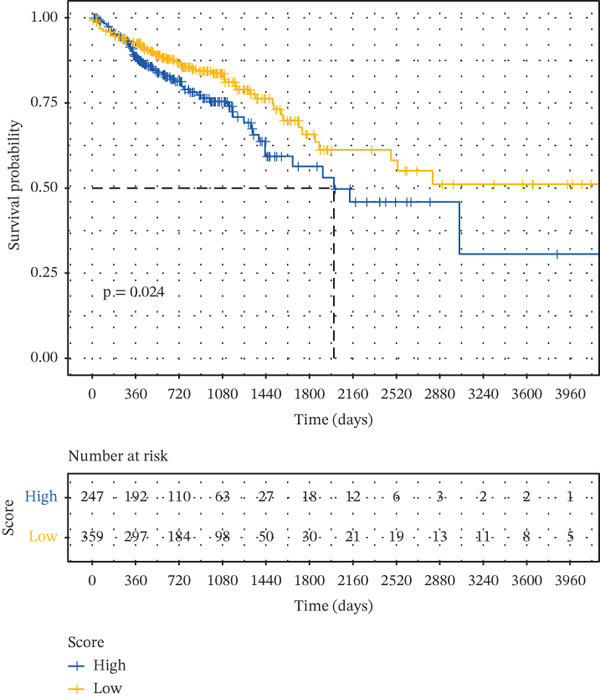
(d)

**Figure:**
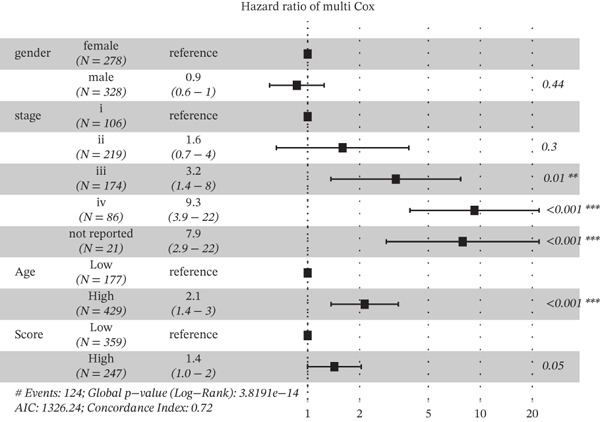
(e)

To assess the robustness of the interworking signature score between subpopulations of tumor cells and subpopulations of CAFs, validation was performed in the independent dataset GSE17534 in GSE39582. We found that the interworking signature score between tumor cell subpopulation and CAF subpopulation has reliable robustness. In GSE39582 in GSE17534, we found that patients with low signature score also had a significant survival advantage and that the signature score of the reciprocal mechanism between tumor cell subpopulations and CAF subpopulations was an independent prognostic factor for CRC (Figures 6a, 6b, 6c, 6d, 6e, and 6f).

Figure 6Validation of signature score for interactions between subpopulations of tumor cells and subpopulations of CAFs in an independent dataset. (a–c) Validation of the robustness of the interworking signature score between subpopulations of tumor cells and subpopulations of CAFs in GSE17534. (a) K‐M curves of the interworking signature score grouping between tumor cell subpopulations and CAF subpopulations. (b) Multivariate Cox forest plot integrating gender, stage, age, and interactions between tumor cell subpopulations and CAF subpopulations for signature score. (c) Heat map of sample distribution and expression levels of six characterized genes in GSE17534. (d–f) Validation of the robustness of the interworking signature score between subpopulations of tumor cells and subpopulations of CAFs in GSE39582. (d) K‐M curves of the interworking signature score grouping between tumor cell subpopulations and CAF subpopulations. (e) Multivariate Cox forest plot integrating gender, stage, age, and interactions between tumor cell subpopulations and CAF subpopulation signature score. (f) Heat map of sample distribution and expression levels of six characterized genes in GSE39582.

**Figure:**
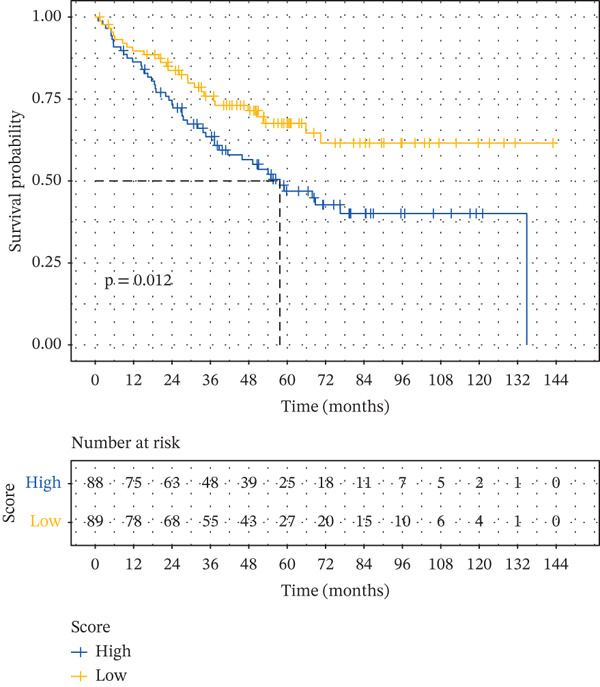
(a)

**Figure:**
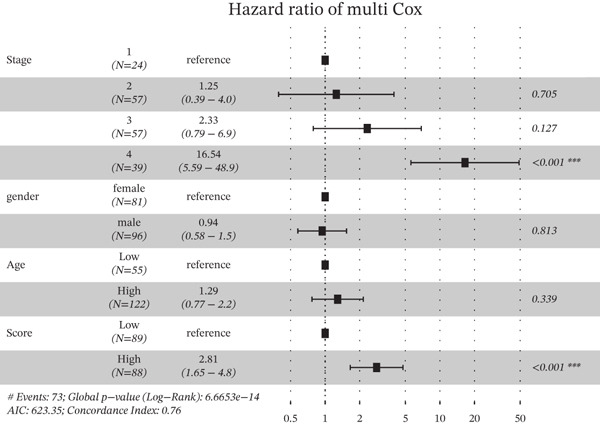
(b)

**Figure:**
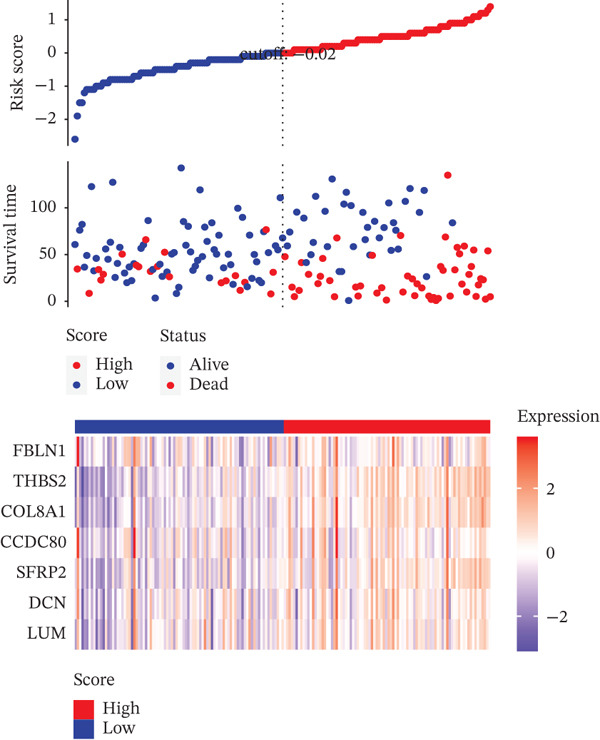
(c)

**Figure:**
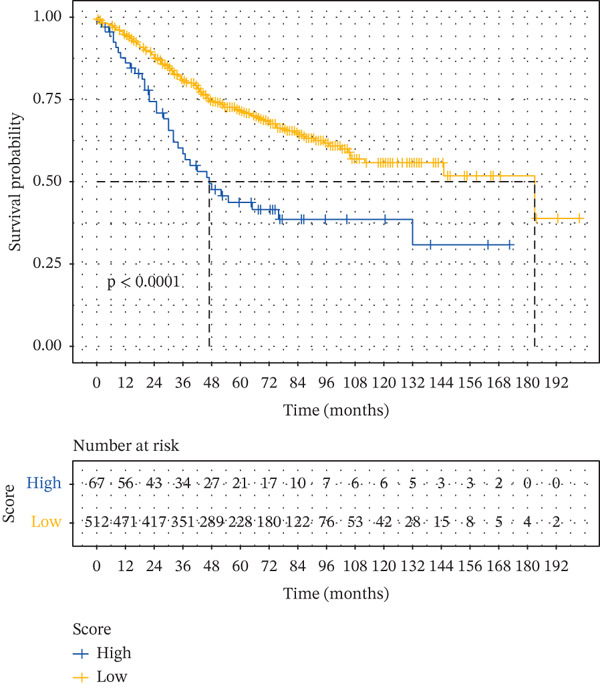
(d)

**Figure:**
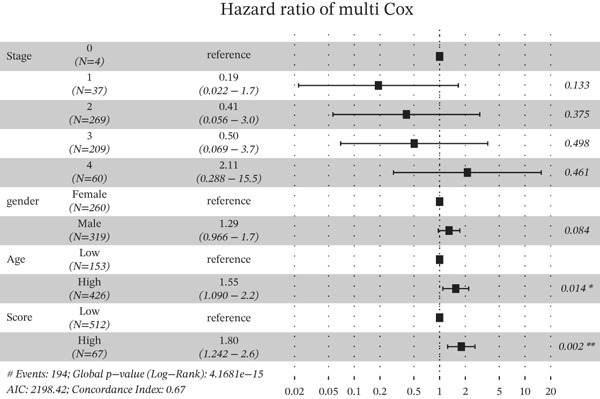
(e)

**Figure:**
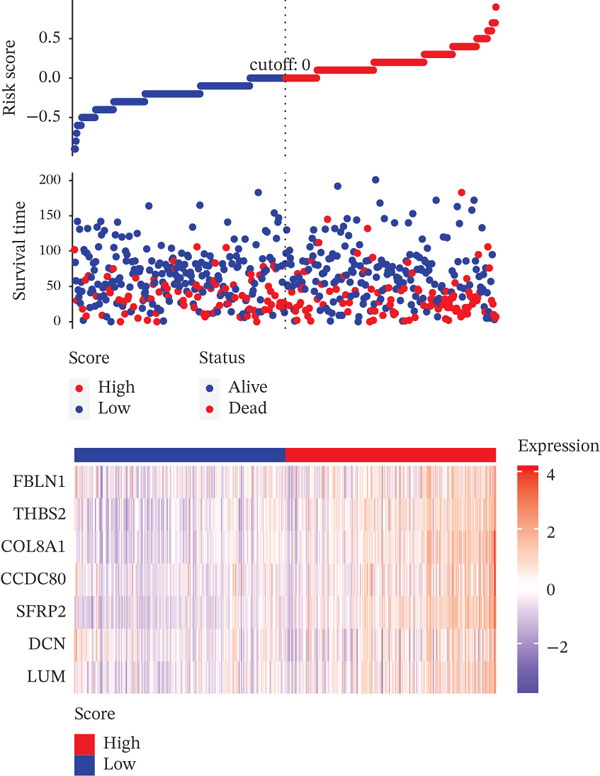
(f)

### 3.6. Interaction Between Subpopulations of Tumor Cells and Subpopulations of CAF Signature Score Predictive of Immunotherapy

Furthermore, we evaluated the predictive performance of the prognostic signature for sample prognosis in the immunotherapy cohort GSE78220 (metastatic melanoma) and GSE42664, which was not statistically significant, but the trend was consistent. In the immunotherapy cohort, again, patients with a high signature score had a poor prognosis, patients in the immunotherapy‐responsive group had a lower signature score than those in the nonresponsive group, and a higher proportion of patients in the low‐score group responded to immunotherapy than those in the high‐score group, suggesting that the low‐score group may be better able to benefit from immunotherapy (Figures 7a, 7b, and 7c).

Figure 7Predictive role of interactions between subpopulations of tumor cells and subpopulations of CAF signature score for immunotherapy. (a) K‐M curves of patients grouped by reciprocal signature score between subpopulations of tumor cells and subpopulations of CAFs in GSE78220. (b) Interaction between subpopulations of tumor cells and subpopulations of CAFs in GSE78220 in PD/SD and CR/PR signature score and proportion of patients in the PD/SD and CR/PR groups. (c) Interaction between subpopulations of tumor cells and subpopulations of CAFs for PD/SD and CR/PR in GSE42664 signature score and proportion of patients in the PD/SD and CR/PR groups.

**Figure:**
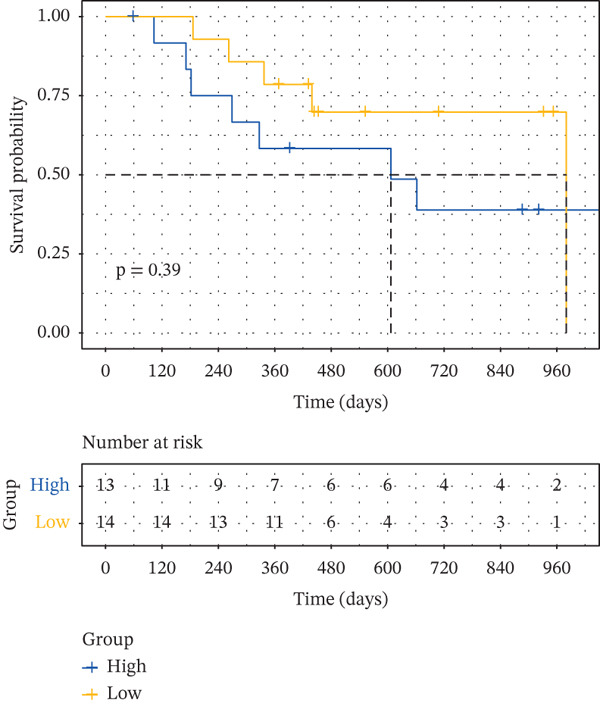
(a)

**Figure:**
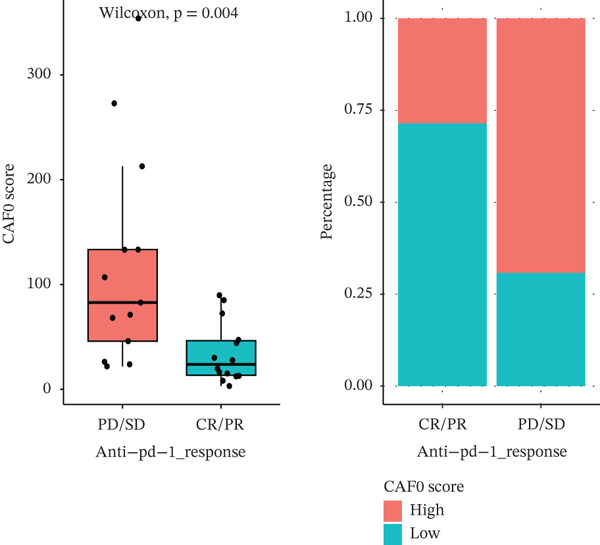
(b)

**Figure:**
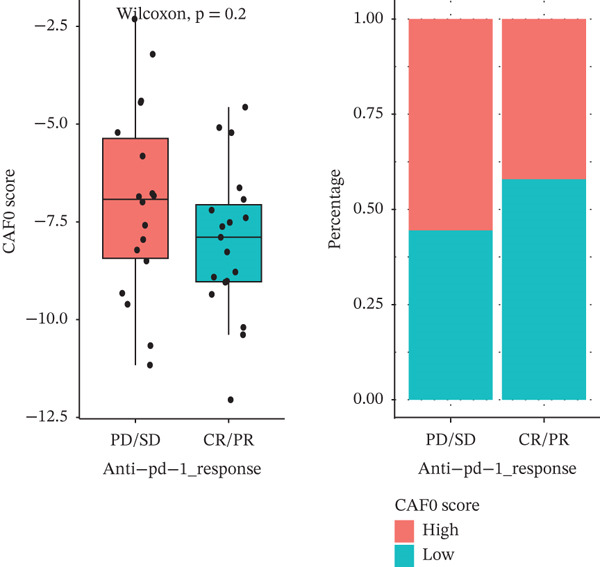
(c)

## 4. Discussion

When CRC patients are diagnosed, some cases have metastatic lesions, and the 5‐year survival rate of metastatic CRC is less than 20%. Currently, chemotherapy is still the best choice for metastatic CRC patients, but the phenomenon of adverse reactions to chemotherapeutic drugs limits the therapeutic effect [28]. Based on single‐cell mapping analysis, cell population differences in tumor tissues can be visualized [11]. The interaction pattern between cells in tumor tissues and tumor‐associated microevirnment (TME) helps to reveal the metastatic mechanism of CRC.

In this study, we first carved out the cell landscape in metastatic CRC and primary CRC. The results showed an increase in fibroblasts and a decrease in tumor cells in metastatic CRC. Based on this phenomenon, we identified a subpopulation of CAF and a subpopulation of tumor cells in them. It was found that CAF interacted with cells in the TME and activated cell signaling pathways by secreting a variety of small molecule bioactive substances, a phenomenon that promotes tumor cells that undergo EMT, growth and invasion, and tumor chemoresistance [29]. In studies of CRC, chemokines produced by CAFs, such as CXCL12 in combination with its receptor CXC4R, can promote tumor proliferation by increasing angiogenesis, and these CAF‐derived chemokines and growth factors have the same synergistic effect on CAFs’ own proliferation and ultimately form a positive feedback loop effect between tumor cells and CAFs, accelerating the progression of CRC [30]. CAFs are also important regulators of CRC metastasis. CAFs secrete growth factors and chemokines and promote TME remodeling. In CRC, CAFs can induce TME remodeling and promote tumor cell metastasis [31]. Another study showed that CAFs were able to trigger the GP130/STAT3 signaling pathway in CRC cells by secreting IL‐11, which in turn promoted CRC metastasis [32]. In addition, IL‐34 derived from CAFs induced normal fibroblasts (NFs) to express *α*‐SMA and FAP, which caused NFs to produce a cell phenotype similar to that of CAFs and functioned to promote tumor metastasis [33]. Furthermore, multiple signaling pathways such as Wnt/*β*‐catenin, Hippo, JAK/STAT, EGFR, and TGF‐*β* are activated in CAFs, which play important roles in the chemotherapy resistance and invasion/metastasis of cancer cells [34]. For instance, activated EGFR signaling cooperates with CAFs to promote the migration of cancer cells [34]. From these studies, it is clear that CAFs are indeed an important factor in inducing CRC metastasis. This was also verified by our results that the proportion of fibroblasts in metastatic CRC was higher than that in primary CRC samples.

Then, based on six characterized genes related to the interaction mechanism between the subpopulation of tumor cells and the subpopulation of CAFs, we constructed a prognostic model of CRC, LUM, DCN, SFRP2, CCDC80, SFRP4, and GREM1. LUM was found to be a characterized gene of CAFs in ovarian cancer, which was closely associated with ovarian cancer prognosis [35]. In CRC, DCN is an important factor in EMT‐related metastatic events [36]. SFRP2 was found to be associated with the proliferation of gastric cancer [37] and inhibited the proliferation and migration of CRC cells [38]. CCDC80 dysfunction in TME is an oncogenic factor in CRC, and in a xenograft model of CRC, CCDC80 dysfunction led to intestinal epithelial organelle formation [39]. The expression level of SFRP4 was associated with very poor OS in CRC patients [40]. GREM1 was found to be expressed in tumor tissues and epithelial cells in CRC patients and was involved in osteoblast differentiation as well as in tumorigenic pathways [41]. Combining these studies, it is easy to see that the six characterized genes we identified related to the mechanism of interactions between the subpopulation of tumor cells and the subpopulation of CAFs are associated with cancer prognosis, progression, and metastasis. This shows that the CRC prognostic model based on the composition of these genes is reasonable. In addition, the prognostic model of CRC demonstrated excellent prognostic value in multiple datasets and had good predictive value for immunotherapy response.

The present study continues to have shortcomings, although we identified characteristic cell subpopulations in metastatic CRC and primary CRC. However, this study was a predictive study based on past data and lacked the validation of cell or animal testing. Based on the results of this study, conducting cell and animal tests to validate the cell heterogeneity and metastatic mechanisms in metastatic CRC and primary CRC makes it an important research goal for us to follow up on. Overall, this study identified the characteristic cell subpopulations in metastatic CRC and primary CRC, and we also constructed a prognostic model of CRC based on the reciprocal genes between them. Our study provides a scientific basis for an in‐depth revelation of the metastatic mechanism of CRC.

## Ethics Statement

The authors have nothing to report.

## Disclosure

All authors read and approved the submitted version.

## Conflicts of Interest

The authors declare no conflicts of interest.

## Author Contributions

All authors contributed to the study conception and design. R.S. and Z.X. contributed to the conception and design of the study. Y.T. organized the database. Y.Y. and F.T. performed the statistical analysis. Y.T., L.H., N.L., M.D., and Y.W. conducted the experiments and analyzed the data. Y.T. and Y.Y. wrote the sections of the manuscript. Y.T., R.S., and Z.X. reviewed the data and finalized the manuscript. All authors commented on previous versions of the manuscript. Y.T. and Y.Y. contributed equally to this work.

## Funding

This study is supported by the Natural Science Foundation of Chongqing (CSTB2025NSCQ‐GPX0297), Municipal Health‐Science Joint Foundation of Chongqing (2026MSXM107), the Research Fund of Sichuan Academy of Medical Sciences, and the Sichuan Provincial People′s Hospital (2023QN05).

## Supporting information


**Supporting Information** Additional supporting information can be found online in the Supporting Information section. Figure S1 Heat map of the expression levels of CAF characterized genes in nine CAF subpopulations.

## Data Availability

The datasets generated during and analysed during the current study are available from the corresponding author on reasonable request.
