# Correction to “Single‐Cell Sequencing Data Revealed Mechanisms of Interactions Between Tumor Cells and Cancer‐Associated Fibroblasts in Metastatic Colorectal Cancer”

**DOI:** 10.1155/bmri/9847386

**Published:** 2026-07-12

Y. Tan, Y. Yang, F. Tian, N. Li, L. Hu, M. Deng, Y. Wang, Z. Xia, R. Sun, “Single‐Cell Sequencing Data Revealed Mechanisms of Interactions Between Tumor Cells and Cancer‐Associated Fibroblasts in Metastatic Colorectal Cancer,” *BioMed Research International*, 2026, 8830690, 10.1155/bmri/8830690

In the article, there was an error in the order of the first two affiliations, leading to an affiliation of Ran Sun being incorrect. The correct author list and affiliations are listed below and have been corrected in the article:

Yiqing Tan^1,2^, Yiping Yang^3^, Fuhua Tian^3^, Ni Li^3^, Lei Hu^3^, Mingyou Deng^3^, Yingying Wang^3^, Zuwei Xia^3^, Ran Sun^2,3^

^1^Department of Breast Surgery, Sichuan Academy of Medical Sciences and Sichuan Provincial People′s Hospital, School of Medicine, University of Electronic Science and Technology of China, Chengdu, China

^2^Key Laboratory of Molecular Oncology and Epigenetics, The First Affiliated Hospital of Chongqing Medical University, Chongqing, China

^3^Department of Oncology, Jiulongpo People′s Hospital, Chongqing, China

We apologize for this error.

